# Anterolateral approach with tibial tubercle osteotomy versus standard medial approach for primary total knee arthroplasty: does it matter?

**DOI:** 10.1186/1471-2474-11-167

**Published:** 2010-07-22

**Authors:** Michael T Hirschmann, Mathias Hoffmann, Robert Krause, Reza-Amir Jenabzadeh, Markus P Arnold, Niklaus F Friederich

**Affiliations:** 1 Department of Orthopaedic Surgery and Traumatology, Kantonsspital Bruderholz, CH-4101 Bruderholz, Switzerland; 2 Musculoskelettal Surgery Department, Imperial College, London, UK; 3 Extremitätenchirurgie/Allgemeine Orthopädie in der Oberlinklinik Potsdam,Rudolf-Breitscheid-Straße 24, 14482 Potsdam, Germany

## Abstract

**Background:**

The purpose of this prospective consecutive multicenter study was to investigate whether the type of surgical approach (medial parapatellar (MPA) or lateral parapatellar with tibial tubercle osteotomy (TubOT)) influences the early clinical and radiological outcomes of primary total knee arthroplasty (TKA).

**Methods:**

Ligament balancing primary TKA with a rotating platform was performed in 143 knees (m:w = 1:1.6; mean age 69 ± 8 years). The TKA was done by a lateral parapatellar subvastus approach with stepcut osteotomy of the tibial tubercle (53%; n = 76, group A) or medial parapatellar approach (47%; n = 67, group B). The outcome was assessed at 1 and 2 years postoperatively by the American Knee Society score (KSS) and the knee society total knee arthroplasty roentgenographic evaluation and scoring system (TKA-RESS). The patient's pain level and satisfaction was noted by a visual analogue scale (VAS). Data were analyzed by an independent statistician with a level of significance of p < 0.05. The Wilcoxon two sample test (two-sided) was used to investigate differences of patients between group A and B pre- and postoperatively. The paired t-test was used to evaluate differences over course of time within each group. For comparison of radiological alignment a Chi^2^-test was performed.

**Results:**

Although having a lower degree of preoperative flexion (112° ± 15° versus 115° ± 15°) patients in group A showed a significantly (p = 0.027) higher degree of flexion (118° ± 10°) at their last follow-up than patients in group B (114° ± 10°). Patients in group A showed a significantly better mean VAS pain (p = 0.0001) and satisfaction (p = 0.0058) at 2 years follow-up. The pain free walking distance was significantly (p = 0.036) longer for group A than group B. Patients treated with a lateral approach were significantly more stable in terms of valgus stress (p = 0.049). The Knee society score was significantly (p = 0.0009) higher at two years follow up in group A compared to group B. The postoperative mechanical alignment and positioning of the prosthesis were not significantly different. Patients in group B presented with significantly (p = 0.0017) more tibial radiolucencies (> 2 mm) at their last follow-up than patients in group A. There was no prosthesis related revision in either group. The revision rate in group A (4%) was higher than in group B (1.5%), which was mainly due to two cases of traumatic secondary displacement of the tibial tubercle and need for refixation.

**Conclusions:**

The TubOT led to slightly better functional results and less pain two years after primary TKA. It is however not clear if the improved outcome can outweigh the longer operation time and higher risk of early complications and revisions. Long-term studies are necessary to show whether there is any difference in prosthesis longevity between both types of approach.

## Background

Total knee arthroplasty (TKA) is a well established orthopaedic surgical intervention in patients with disabling primary tri-compartmental osteoarthritis [1-10]. Although many studies have investigated the influence of the chosen surgical approach on outcome there remains its controversy[1,2,11-35]. Most authors use a medial parapatellar arthrotomy[8,10,34,36] (MPA), which in whatever variation used, may possibly lead to painful scarring and reduced strength of the extensor apparatus[25,37,38]. Limited surgical exposure may further complicate correct ligament balancing and implant positioning[13]. By the use of a lateral parapatellar subvastus approach with tibial tubercle osteotomy (TubOT) the quadriceps muscle is preserved and the extensor mechanism and vascular supply of the patella is maintained[39]. However, this approach has not gained much acceptance in primary total knee arthroplasty[17,35,39], whereas it has in revision surgery[40-44]. Most surgeons consider it to be technically more demanding, time consuming, and associated with a higher risk of complications than other approaches. Just recently Piedade et al.[45] reported no differences in terms of functional outcomes between medial parapatellar and lateral parapatellar approach with bevelcut tibial tubercle osteotomy using an oscillating saw. However, in this study the tibial tubercle osteotomy was associated with complications such as skin necrosis and fracture of the tibial tubercle and in their opinion it should therefore only be performed in cases when an adequate surgical exposure is restricted with a medial parapatellar approach.

The purpose of this prospective consecutive multicenter study was to investigate if the type of surgical approach, whether medial parapatellar or lateral parapatellar subvastus approach with tibial tubercle osteotomy, influences the early clinical and radiological outcomes of ligament balancing primary total knee arthroplasty.

## Methods

**Figure 1:**
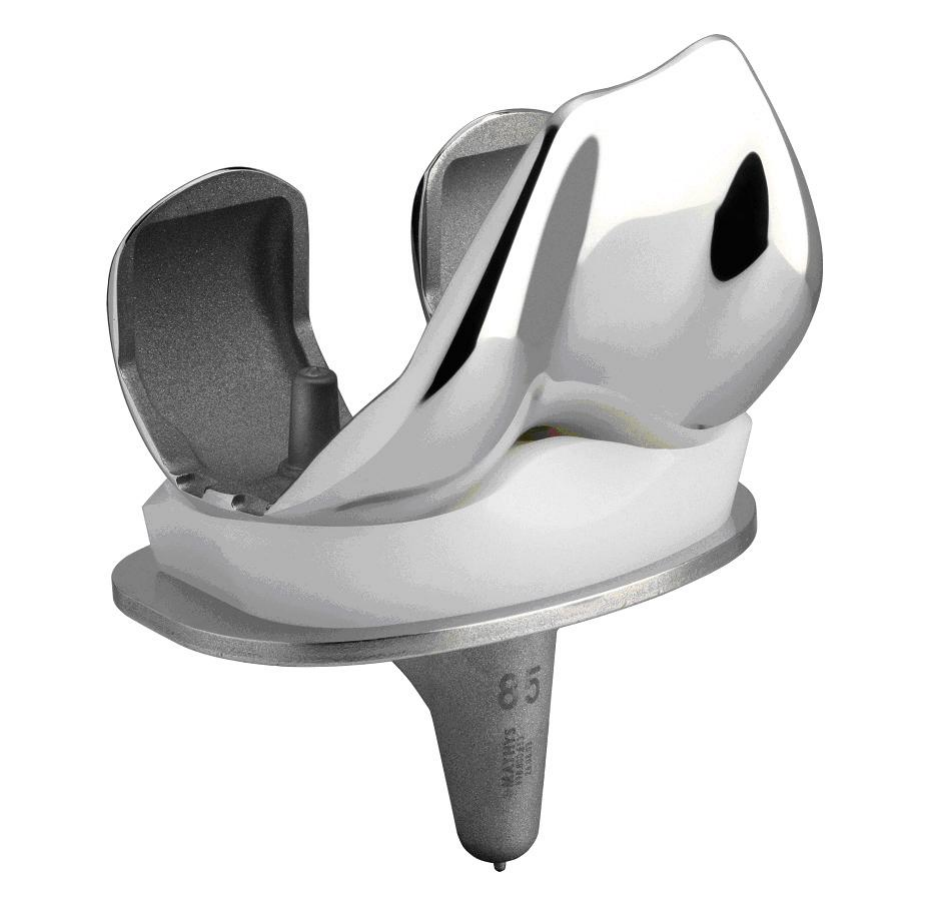
Bicondylar ligament cruciate retaining balancing total knee arthroplasty with rotating platform (balanSys^®^, Mathys Ltd., Bettlach, Switzerland)

From January 2005 to January 2007 136 (unilateral n = 126, bilateral n = 10) consecutive patients were treated with a primary, ligament balancing, posterior cruciate retaining total knee arthroplasty (balanSys^®^, Mathys Ltd, Bettlach, Switzerland - fig.1) due to tri-compartmental osteoarthritis of the knee joint. Three patients were lost to follow-up, resulting in 133 patients (143 knees) with a complete follow-up time of two years after the operation (mean follow up time 25 ± 4 months, follow-up rate 98%).

Of these 143 knees, 76 (53%) had a TubOT-approach (group A) and 67 (47%) had a MPA approach (group B).

The decision for the approach used was based on surgeon's preference. The patients' characteristics of both groups are presented in table 1.

**Table 1:** Demographic data of patients between groups investigated

	Group A	Group B	Significance
**Number of patients**	n = 76	n = 67	-

**Mean age at surgery in years ± standard deviation**	72 ± 8	67 ± 7	p = 0.0001

**male/female**	33/43	22/45	n.s.

**Mean body mass index (BMI) ± standard deviation (range)**	29 ± 5 (20-42)	31 ± 5 (22-42)	p = 0.012

**Mean follow up time ± standard deviation**	25 ± 3	26 ± 5	n.s.

Wilcoxon Two Sample Test (two sided), level of significance p < 0.05

The surgery was performed by experienced surgeons at three knee centres; a university affiliated teaching hospital and two regional hospitals. In one center the lateral Tub-OT approach was performed. The MPA approach was performed in all study centers. All patients were routinely informed about the operation, the use of the prosthesis and agreed to participate according to the protocol. Informed consent was obtained from all patients in accordance with the institutional review board at our institution.

### Operative technique

In group A all patients had a "Bruderholz" technique approach (TubOT)[39] and in group B a medial parapatellar approach (MPA)[46] was performed. All interventions were performed by experienced surgeons specialized or specializing in orthopaedic surgery.

**Figure 2:**
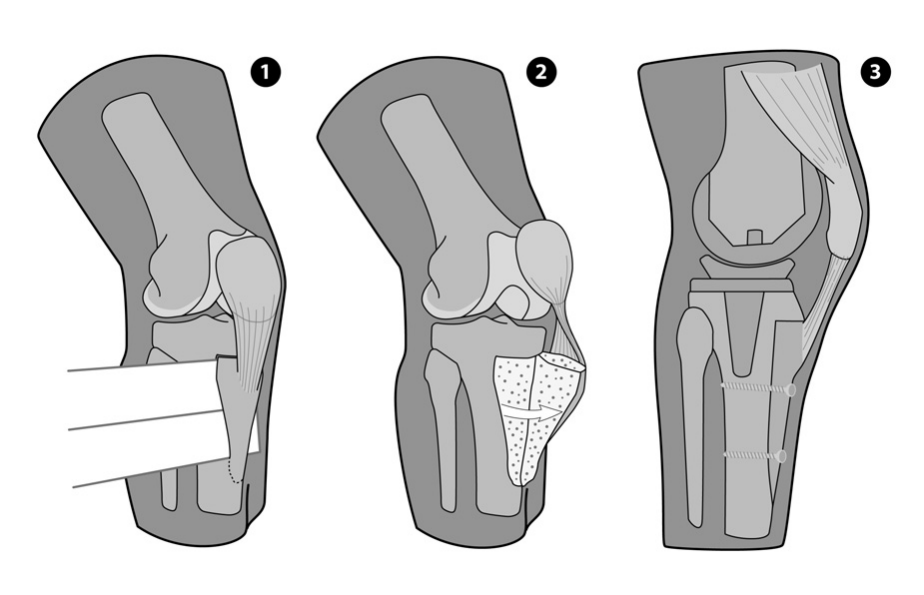
Schematic image of the stepcut tibial tubercle osteotomy

For the "Bruderholz" approach a step cut at the proximal end of the osteotomy was made with a thin osteotome before completing the osteotomy with different sized osteotomes to provide resistance against proximal displacement. The distal part of the quadriceps tendon, the patella, the patellar ligament, and the tibial tuberosity were retracted medially taking care not to detach the periostium bridge of the medial side of the tibial tubercle. Two 3.5 mm cortical screws were used as lag screws for refixation of the tibial tubercle (fig.2).

The tibial component was cemented in all patients, the femoral component in 75 patients (60%). In six knees (4%) the patella was resurfaced in all other knees it was denervated with electrocautery. A tourniquet (350 mmHg) was used for all operations and deflated before wound closure. The surgical procedure was in every case identical except for the individual size of the components and the completely different surgical approach.

**Figure 3:**
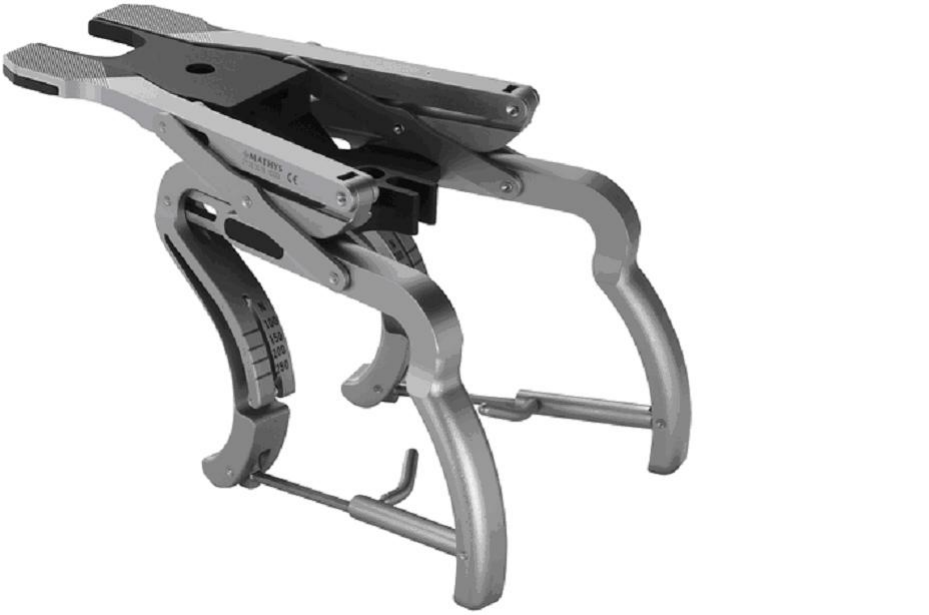
Ligament tension referencing system with a double spring tensor for optimal ligament balancing

For ligament balancing of the knee joint a double spring tensor with Newton scale was used, which is able to separately address tension of the medial and lateral joint compartment (fig. 3). In full extension a load of 150-200 N was applied and the ligament tension subsequently modified by lateral or medial release. The same procedure was performed in 90° flexion and 100 N applied. As described by Peters[47] extension structures were released when knee was tight in extension and flexion structures were released when the knee was tight in flexion.

An intramedullary jig was used for femoral bone cuts (5° or 7° valgus), for tibial bone cuts were aligned using an extramedullary jig. Drains were placed for 48 hours postoperatively. Antibiotic prophylaxis and low molecular weight heparin for prevention of deep vein thrombosis were used.

Postoperatively active assisted physiotherapeutic exercises starting on the first day with unrestricted active motion and weight bearing were encouraged.

### Follow-up

The prospective follow-up regimen consisted of standardized clinical as well as radiological evaluations preoperatively, 12 months and 24 months postoperatively.

The patients were asked whether they have any knee pain and when present if this was related to climbing stairs, climbing stairs and walking, or kneeling. Their pain free ability to walk was graded (unlimited; 30-60 minutes > 2000 meters; 15-30 minutes 1000-2000 meters; < 15 minutes < 1000 meters; only inside the home; impossible).

In addition, the patients rated their pain level and perceived satisfaction on a visual analogue scale - VAS (min. 0 - max.10).

On clinical examination active and passive range of motion (flexion and extension) were measured using a goniometer. The anterior laxity of the knee joint was measured using the Rolimeter (Ormed, Freiburg, Germany). The valgus/varus laxity was assessed clinically in 30° flexion (< 5°, 5°-10°, 10°-15°, > 20°). Functional evaluation included the scoring system of the American Knee Score (KSS)[48]. The KSS is a well established and one of the most frequently used outcome instrument for evaluating patients after total knee arthroplasty, which consists of the Function Score (0-100), the Knee Score (0-100) and the Total Knee Score-KSS (0-200)[48,49].

**Figure 4:**
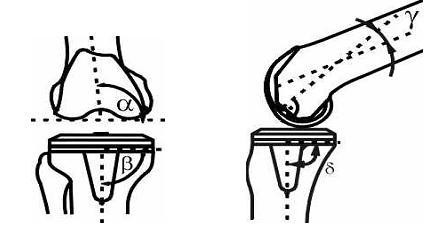
Implant position (femoral flexion angle α, the tibial angle β, the femoral angle γ and the tibial angle δ)

For radiological evaluation standardized weight-bearing anteroposterior, true lateral radiographs, skyline view and long leg radiographs were used. Two of the authors not involved in the index procedures examined the radiographs with respect to "The knee society total knee arthroplasty roentgenographic evaluation and scoring system (TKA-RESS)[50]". The radiographs were analysed for implant position, radiolucency and mechanical alignment. In anteroposterior radiographs the femoral flexion angle α and the tibial angle β were measured. In lateral radiographs the femoral angle γ and the tibial angle δ were measured (fig. 4). Pre- and postoperative leg alignment was recorded on long leg radiographs.

Complications such as skin necrosis, delayed or non-unions of the osteotomy site, extensor lag, a tibial plateau fracture, a displacement of the tibial tubercle, limited flexion < 90° and need for secondary surgeries were recorded.

The rates of prosthesis survival at two years follow up for the endpoint - revision for any prosthesis related reason- and revision for any complication- were assessed.

### Statistical methods

Mean, median, standard deviation and range were reported for continuous variables and relative and absolute frequencies for categorial variables.

The Wilcoxon two sample test (two-sided) was used to investigate differences of patients between group A and B (gender, number of previous surgeries, date of surgery, duration of hospital stay, mean age at surgery, body mass index (BMI), operation time).

A confounder adjustment with random effects models was performed to analyze the influence of age and BMI on the difference in outcome scores.

The Wilcoxon two sample test (two-sided) was also used to investigate differences between group A and B postoperatively (VAS pain, VAS satisfaction, pain free walking distance, ability of pain free stair climbing, anterior and valgus/varus laxity, Function score, Knee Score, Total KSS). The paired t-test was used for the aforementioned postoperative variables to evaluate differences over course of time within each group. For comparison of radiological alignment a Chi^2^-test was performed.

Data were analyzed using SAS statistical analysis package (Cary, North Carolina, USA). The level of significance was defined as p < 0.05.

## Results

### Patients and interventions

Both treatment groups did not differ significantly in terms of gender, number of previous surgeries, date of surgery and duration of hospital stay (table 1). Patients in group A (72 ± 8 years) were significantly (p < 0.001) older than in group B (67 ± 7 years). The BMI in group B (31 ± 5) was significantly (p = 0.012) bigger than in group A (29 ± 5). The operation time was significantly (p = 0.0001) longer in group A (116 ± 20 minutes) than group B (89 ± 22 minutes). A confounder adjustment with random effects models did not show any influence of age and BMI on the difference in outcome scores.

The preoperative alignment differed significantly (p = 0.0018) between group A and B. This is presented in table 2.

**Table 2:** Comparison of the pre- and postoperative mechanical alignment between groups investigated

	Preoperative		Postoperative	
	**Group A**	**Group B**	**Group A**	**Group B**

**Neutral**	21 (28%)	12 (18%)	73 (96%)	62 (92.5%)

**Varus**	39 (51%)	52 (78%)	3 (4%)	3 (4.5%)

**Valgus**	16 (21%)	3 (4%)	-	2 (3%)

**Significance**	p = 0.0018		n.s.	

Chi2 test, level of significance p < 0.05

The median duration of hospital stay was 13 ± 3 days with a non-significant difference between group A (13.3 ± 3.4 days) and B (12.5 ± 2.2 days). 46% (n = 66) of patients had undergone a previous operation before total knee arthroplasty. Eleven patients had undergone an arthroscopy, eleven a partial meniscectomy, six a high tibial osteotomy and 17 any other knee surgery. There was no significant difference between both groups. The mean operation time in group A was 116 ± 20 minutes and 89 ± 22 minutes in group B.

### Clinical and radiological outcome

In group A 70% of knees (n = 53) were pain free with any activity, in group B 57% (n = 33). The pain free walking distance was significantly (p = 0.036) longer for group A than group B. In group A (n = 56 74%) there were significantly more patients than in group B (n = 34 51%), which had an unlimited walking distance at last follow-up. By trend (p = 0.066) more patients in group A (n = 52 69%) were able to climb stairs without any impairment than in group B (n = 33 50%). Patients in group A showed a significantly better VAS pain (p = 0.0001) and satisfaction (p = 0.0058) at 2 years follow-up (table 3).

**Table 3:** Outcome preoperative, 12 and 24 months after surgery (mean ± standard deviation) for group A and group B

Outcome	Before surgery		Significance	12 months after surgery		24 months after surgery		Significance		
	**Group A**	**Group B**	**Between groups**	**Group A**	**Group B**	**Group A**	**Group B**	**Between groups**	**Within group A**	**Within group B**

VAS pain	6.9 ± 1.4	7.0 ± 1.3	n.s.	1.5 ± 1.9	1.7 ± 1.9	0.9 ± 1.7	1.4 ± 1.7	p < 0.0001	p < 0.0001	p < 0.0001

VAS satisfaction	3.8 ± 1.8	3.5 ± 2.0	n.s.	8.8 ± 1.6	8.1 ± 1.9	9.1 ± 1.6	8.5 ± 2.2	p = 0.0059	p < 0.0001	p < 0.0001

Flexion passive	112 ± 15	115 ± 15	n.s.	115 ± 11	114 ± 11	118 ± 10	114 ± 10	p = 0.020	p = 0.0017	n.s.

Knee Score (KSS) Total	103 ± 25	86 ± 26	p = 0.00014	180 ± 24	166 ± 26	182 ± 25	171 ± 27	p = 0.0008	p < 0.0001	p < 0.0001

KSS - Knee Score	50 ± 15	40 ± 15	p = 0.00024	91 ± 11	86 ± 14	93 ± 11	88 ± 13	p = 0.0004	p < 0.0001	p < 0.0001

KSS - Function Score	53 ± 17	46 ± 19	p = 0.027	90 ± 17	81 ± 16	89 ± 18	83 ± 18	p = 0.015	p < 0.0001	p < 0.0001

Wilcoxon Two Sample Test (two sided) for differences between groups, paired t-test for differences within group, level of significance p < 0.05

Although having a lower degree of preoperative flexion (112° ± 15° versus 115° ± 15°) patients in group A (118° ± 10°) showed a significantly (p = 0.027) higher degree of flexion at last follow-up than patients in group B (114° ± 10°).

The anterior laxity using the Rolimeter showed no significant difference between group A and B with 97% of patients having a < 5 mm anterior-posterior translation. Patients treated with a lateral approach were significantly more stable in terms of valgus laxity (p = 0.049). In group A 82% of patients (n = 62) and in group B 64% of patients (n = 43) had a valgus laxity < 5°. The pre- and postoperative mechanical alignment in both groups is presented in table 2.

The Knee society score was significantly higher at two years follow up in group A compared to group B. The course over time is presented in table 3. Similar results were found for the Knee and Function score.

The femoral angle α (varus-valgus) at last follow-up was significantly (p = 0.021) different in group A 90° ± 7° and group B 87° ± 8°. A similar significant (p < 0.001) difference was found in terms of the tibial angle β (group A 91° ± 2°, group B 89° ± 2°), which indicates the varus-valgus alignment of the prosthesis. The femoral angle γ indicates flexion/extension of the femoral component and showed no significant difference between both groups (group A 85° ± 4°, group B 85° ± 4). The tibial angle δ (tibial inclination) showed no significant difference between both groups (group A 85° ± 4°, group B 85° ± 4).

There were no differences in radiolucencies of the femoral site between both groups according to the knee society total knee arthroplasty roentgenographic evaluation and scoring system (TKA-RESS). Patients in group B presented with significantly (p = 0.0017) more tibial radiolucencies (> 2 mm) at last follow-up than patients in group A.

### Complications (table 4)

No local complications e.g. skin necrosis occurred. No delayed or non-unions of the osteotomy site occurred. No extensor lag was present in both groups. In one patient (group A) a tibial plateau fracture occurred intraoperatively, which was treated with a cast and partial weight bearing for 6 weeks. In two patients of group A the tibial tubercle secondarily displaced in two cases and was subsequently reattached. In one patient with arthrofibrosis and limited flexion < 90° (group A) an arthroscopic arthrolysis was successfully performed.

**Table 4:** Major adverse events and revision surgery for patients in group A and B

No.	Name	Group	Age	Gender	BMI	Adverse event	Revision surgery	VAS pain preop	VAS satisfaction preop	KSS preop	VAS pain last FU	VAS satisfaction last FU	KSS last FU
1	M.E.	A	73	F	35	Posttraumatic displacement of tibial tubercle	4 weeks p.o. refixation	8	2	80	2	8	188

2	J.R.	A	67	F	26	Proximal migration of tibial tubercle	3 weeks p.o. refixation	8	4	88	2	9	192

3	V.L.	A	62	F	22	Flexion < 90°	Arthrolysis	6	3	124	0	7	188

4	MG	B	68	F	29	progression of patellofemoral disease	Resurfacing patella	7	-	87	5	7	126

In one patient of group B with progress of patellofemoral osteoarthritis and new onset of anterior knee pain the patella was secondarily resurfaced.

The estimated rate of prosthesis survival at two years follow up for the endpoint - revision for any prosthesis related reason - was 100% in group A and 100% in group B. With the endpoint - revision for any complication - the estimated rate of survival was 96% in group A and 98.5% in group B.

## Discussion

The type of surgical approach used for primary total knee arthroplasty is considered to importantly influence postoperative outcomes[12-14,16,17,24-26,30,39]. Hence, we wanted to investigate the influence of two different types of approaches (MPA versus TubOT) on early clinical and radiological outcomes in patients undergoing ligament balancing primary total knee arthroplasty. To the best of our knowledge this is the only study comparing these two approaches. The major implications of this prospective study are threefold:

First, patients treated with a lateral approach with TubOT showed significantly better functional results, less pain and more satisfaction two years after total knee arthroplasty than patients treated with a medial parapatellar approach.

Second, patients treated with a medial approach presented with significantly more tibial radiolucencies at last follow-up than patients treated with a lateral approach. No differences in prosthetic or mechanical alignment were found.

Third, we were obliged to observe an overall revision rate of 4% in patients treated with a lateral approach and 1.5% in patients treated with a medial approach respectively. The most frequent cause for revision surgery was not implant-related, but due to a secondary proximal migration of the tibial tubercle after tibial tubercle osteotomy.

Compared to others reporting results of patients after TKA the functional results and Knee society score at last follow up are comparable[10,45,51,52]. The better functional results in terms of the Knee society score for patients treated with a lateral parapatellar approach might be partly explained by the higher preoperative score values. This is also true for the pain and satisfaction level. However, although the preoperative flexion was worse we found a significantly higher flexion rate for this group, even though the difference was small and hardly of clinical relevance.

The only difference in radiological evaluation between both groups was that patients in group B presented with significant more tibial radiolucencies at last follow-up than patients in group A, which is a result that should be further followed up. To date this finding had no clinical impact on outcomes. Good implant position was achieved with both approaches. No differences in prosthetic or mechanical alignment were found.

Traditionally it is the philosophy of one of the study hospitals to routinely perform a stepcut osteotomy of the tibial tubercle in combination with the lateral parapatellar approach for primary TKA. The utility and need of this approach in primary total knee arthroplasty is a matter of controversy[44,53,54]. Conceptual advantages are a wide exposure of the knee joint with direct visualization of the pertinent anatomical structures. Also minimal tension on the extensor mechanism is applied during eversion of the patella and the medial vascular supply of the patella is preserved. An optimal rotational orientation of the implants and a stepwise soft tissue balancing is facilitated, which might directly influence outcome in total knee arthroplasty[44].

However, many authors are concerned about the higher risk for early complications such as skin necrosis, tibial tubercle displacement or migration, tibial plateau fracture, non or delayed union and extensor lag[40,44,45,55]. For instance this variety of approach related complications was only partially reflected by our data. Neither local complications e.g. skin necrosis, delayed or non-union of the osteotomy site nor an extensor lag occurred, a finding we attribute to a meticulous and careful surgical osteotomy technique. However, one case of a tibial plateau fissure and two cases of proximal migration of the tibial tubercle are clearly approach related complications.

The two cases of postoperative proximal migration of the tibial tubercle could be explained in one case due to an insufficient proximal step-cut after cutting of the proximal tibia for the TKA and in the other case due to a fall on the knee. Interestingly the two patients although having undergone a refixation of the tibial tubercle, in none of these cases the complication contributed to a compromised outcome. Both presented with low pain levels, were highly satisfied and reported good knee scores.

Strategies to prevent these complications are according to Wolf et al. a proper patient selection, as rheumatoid arthritis and a history of at least one previous operation about the knee were predisposing factors for these complications[56]. In our series we could not confirm this finding. The correct sizing of the tibial tubercle fragment[44], which should be 7-8 cm in length; 2 cm in width and 1 cm in thickness, and a proximal step-cut barrier are also reported to be of paramount importance to prevent such complications. In our experience the proximal step-cut has an important function as abutment against the quadriceps forces pulling in proximal direction[39]. In combination with the used two lag screws the distinct contact area of the tibial tubercle fragment with the tibia provided a safe condition for direct bone healing[39].

One could speculate that the only complication in the MPA group a single case of progression of the patellofemoral disease may be attributed to the medial parapatellar approach leading to an altered patella tracking and higher pressure on the patellofemoral joint, but this would overestimate the importance of the results. However, a good patella tracking is considered to be a major advantage of the TubOT approach[39].

Clearly, the higher early revision rate was approach and not prosthesis related. Another important disadvantage of the lateral TubOT was the longer operation time, which may be a reason for the low acceptance of the lateral approach with tibial tubercle osteotomy.

With the trend for minimally invasive approaches some authors would call the TubOT approach for primary TKA an anachronism, which might be correct if the only defining variable is the length of the skin incision. With the TubOT less soft tissue tension is applied, the extensor mechanism and the vascular supply of the patella are preserved and a wide exposure is gained - is this not the meaning of minimal invasiveness? A minimal invasive approach should not be defined by the length of the skin incision, but by the soft tissue handling in deeper layers. In contrast to our study, in which no skin necrosis occurred several studies investigating standard or minimal invasive techniques reported high rates of skin necrosis[57].

We acknowledge several limitations to our study. Patients of both treatment groups were selected by surgeon's preference and not by random, which is reflected by the slightly different patient's characteristics (age, BMI). The radiographs were performed in a standardized manner with careful attention to patient positioning, particularly rotation, but not in every case fluoroscopy controlled. Hence, the accuracy of the radiological measurements is limited. The influence of the prosthesis, particularly the rotating bearing, on outcomes in both groups was not evaluated, but may represent an important factor, as it promises not only a reduced polyethylene wear by less loading stresses transmitted to the inlay, but also offers an increased component conformity and better compensation of rotational malalignment.

Despite these limitations in our opinion there is sufficient evidence, to conclude that the lateral parapatellar approach with tibial tubercle osteotomy led to at least comparable functional results and less pain after total knee arthroplasty at two years follow-up. The burning question however remains if this can outweigh the higher risk of early complications and revisions. Long-term studies are necessary to show whether there is any difference in prosthesis life time between both types of approach.

## Conclusions

The lateral parapatellar approach with tibial tubercle osteotomy led to slightly better functional results and less pain two years after primary TKA. It is however not clear if the improved outcome can outweigh the longer operation time and the higher risk of early complications and revisions. Long-term studies are necessary to show whether there is any difference in prosthesis longevity between both types of approach.

## Competing interests

The study was supported by a financial grant from Mathys Ltd., Bettlach, Switzerland. The external funding source did not have an influence on the investigation.

## Authors' contributions

MH set up the protocol, organized ethics approval, carried out the study and drafted the manuscript. MH participated in the design of the study, the clinical and radiological follow-up and helped with the analysis of radiological data. RK participated in the design of the study and clinical follow-up. RAJ and MPA helped with the data analysis and draft of the manuscript. NFF participated in the design of the study, interpretation of the results and helped with the draft of the manuscript. All authors read and approved the final manuscript.

## Pre-publication history

The pre-publication history for this paper can be accessed here:

http://www.biomedcentral.com/1471-2474/11/167/prepub
